# Editorial: Structure and Function of Chloroplasts

**DOI:** 10.3389/fpls.2018.01656

**Published:** 2018-11-14

**Authors:** Rebecca L. Roston, Juliette Jouhet, Fei Yu, Hongbo Gao

**Affiliations:** ^1^University of Nebraska-Lincoln, Lincoln, NE, United States; ^2^UMR5168 Laboratoire de Physiologie Cellulaire Vegetale (LPCV), Grenoble, France; ^3^Northwest A&F University, Xianyang, China; ^4^Beijing Forestry University, Beijing, China

**Keywords:** chloroplast, photosynthesis, lipid, envelope, thylakoid, development

The primary energy resource of life on earth is the sun, whose energy is captured in the form of usable carbons by a process called photosynthesis. Photosynthesis occurs within a cellular organelle adapted to that purpose, called the chloroplast. Chloroplasts are unique metabolic and sensory organelles restricted to plants, algae, and a few protists. In this special topic, we aimed to gather new research, hypotheses, and reviews that would help us to better understand the important role of chloroplasts in all photosynthetic organisms. We were fortunate enough to have submissions from many talented chloroplast researchers. This topic contains a total of 24 papers of which 13 are original research, 3 are methods, 5 are reviews or mini-reviews, 2 are perspectives and one is a hypothesis.

Chloroplasts developed in an ancient eukaryotic symbiosis, which has since evolved into many divergent organisms. Because of this, studies on algae and other early endosymbionts are particularly relevant as they help us to triangulate back to a better understanding of that first endosymbiotic event. From *Euglena gracilis*, a unique protist with an evolutionarily recent algal endosymbiont, the importance of balancing energy needs with organelle abundance was demonstrated (Shibata et al.) From the model green alga species *Chlamydomonas reinhardtii*, are included three advances. First, a protein conserved in green lineages and diatoms was found to regulate chloroplast redox and be important for the accumulation of Photosystem II (PSII; Xing et al.) Second, a ferredoxin-dependent biliverdin reductase was discovered to function both in retrograde bilin biosynthesis and interaction with light-dependent protochlorophyllide oxidoreductase (Zhang et al.) Third, a new method to isolate intact chloroplasts after nitrogen stress as monitored by ultrastructure observation and fatty acid biomarkers analysis was developed (Yang et al.)

Chloroplasts make many major metabolic contributions to the cell. Photosynthesis has been studied for many decades, but the finer details remain to be established. Recent advances in single-particle cryo-electron microscopy, X-ray free electron laser, and other techniques have revealed unprecedented structural and catalytic details of the photosynthetic protein complexes, which are reviewed by Gao et al. with an emphasis on the light-harvesting complex of PSII. Chlorophyll fluorescence imaging is one of the most important tools for studying photosynthesis. Ruhle et al. reviewed various chlorophyll fluorescence-based genetic screening strategies in *Arabidopsis* and *Chlamydomonas*. Type 1 NADPH dehydrogenase is a protein interacts with Photosystem I (PSI) and has a known role in respiration, cyclic electron transport around PSI and CO_2_ acquisition. This protein was also shown to be important for stabilizing the PSI complex in *Synechocystis* under stress conditions by Zhao et al. Other chloroplast metabolic outputs covered by this special issue include vitamin E/tocopherol and its precursors which is reviewed by Pellaud and Mene-Saffrane and the role of vitamin E/tocopherol in phosphate stress tolerance which was probed by Allu et al. The assembly and transfer of iron-sulfur clusters critical for many aspects of plant metabolism was reviewed by Lu. An important methodological advance in generating cell suspension cultures with which to investigate photosynthesis and other chloroplast metabolic outputs was provided by Sello et al.

Chloroplasts have a number of structures that allow photosynthesis to occur. One of these is the photosynthetic (thylakoid) membrane, which is composed primarily of galactolipids. The role of galactolipids in chloroplast biogenesis was nicely reviewed by Rocha et al. Kobayashi et al. added their perspective on thylakoid phosphatidylglycerol content, and Phosphatidic Acid Phosphohydrolases were shown to be critical for the maintenance of chloroplast membranes (Yoshitake et al.) A natural consequence of positioning highly desaturated membrane lipids near to highly-reactive photosynthetically-derived reactive oxygen species is the production of oxylipins, a subset of which are highly toxic. These can be reduced by the chloroplast envelope quinone oxidoreductase homolog, and its structure was solved by Mas et al. A perspective on the extent to which a thylakoid metalloprotease called FtsH2 controls reactive oxygen signaling was contributed by Dogra et al. An outstanding question in the field of thylakoid structure is how the thylakoid membranes are developed, and a new mechanistic hypothesis regarding the involvement of dynamin-like proteins was contributed by Jilly et al.

The photosynthetic thylakoid membranes are encapsulated within the chloroplast double envelope membranes. All of these must divide together to propagate, and the gene *GIANT CHLOROPLAST 1* was previously suggested to play a critical role in this process. However, Li et al. used CRISPR-mediated gene editing to show that it rarely has giant chloroplasts and likely only has a minor role in chloroplast division. Interestingly, Giant Chloroplast 1 is an inner envelope protein well conserved in plants and its true function awaits further investigation. Encapsulated by the envelopes is a special environment called the stroma which has a strongly variable pH, due to the movement of protons during photosynthesis. Su and Lai established a simple and non-destructive method to measure the stromal pH using a fluorescent dye. Several other genes affecting chloroplast development are reported. The first was previously known only through its mutant phenotype as *variegated5-1*. Its function was defined as a likely Mg^2+^ transporter by Liang et al. leading to interesting new avenues to investigate the role of Mg in chloroplast development. The role of Mg in chlorophyll biosynthesis is well known and was expanded in soybean by Zhang et al. where a complex regulatory system may be needed to control Mg incorporation into chlorophyll. The second, *Dwarf and Yellow 1* may represent a new regulatory mechanism of chloroplast biogenesis and was reported by Huang et al. The third, *Shikimate Kinase-Like 1*, which seems to lack function in the shikimate pathway, was shown to be important for chloroplast development and also to be involved in auxin-related pathways by Xu et al. The majority of the above studies rely on the excellent genetic systems available in model species. In some non-model plants, like the peach tree *Prunus persica*, chloroplast development varies widely by the highly defined tissue types. The strong divergence of the tissues was taken advantage of by Chen et al. which mapped the roles of transcription factors in controlling tissue-specific development. Clearly, further studies are needed to uncover all of the critical components of chloroplast biogenesis.

Together, the papers span a wide breadth of chloroplast structure and function advances from investigations of photosystem structures and the membranes on which they reside, to development within non-model protists and trees. It is our hope that these exciting advances prompt research into the new avenues of research begun here.

## Author contributions

All authors listed have made a substantial and intellectual contribution to the work, and approved it for publication.

### Conflict of interest statement

The authors declare that the research was conducted in the absence of any commercial or financial relationships that could be construed as a potential conflict of interest.

